# Efficacy of Conversion Surgery Following Apatinib Plus Paclitaxel/S1 for Advanced Gastric Cancer With Unresectable Factors: A Multicenter, Single-Arm, Phase II Trial

**DOI:** 10.3389/fphar.2021.642511

**Published:** 2021-03-19

**Authors:** Zhiyuan Xu, Can Hu, Jianfa Yu, Yian Du, Ping Hu, Guofa Yu, Conggang Hu, Yu Zhang, Wei Mao, Shanqi Chen, Xiangdong Cheng

**Affiliations:** ^1^ Department of Gastric Surgery, Institute of Cancer Research and Basic Medical Sciences of Chinese Academy of Sciences, Cancer Hospital of University of Chinese Academy of Sciences, Zhejiang Cancer Hospital, Hangzhou, China; ^2^ The 2nd Clinical Medical College, Zhejiang Chinese Medical University, Hangzhou, China; ^3^ Department of Gastrointestinal Surgery, the First Affiliated Hospital of Zhejiang Chinese Medical University, Hangzhou, China; ^4^ Department of Gastrointestinal Surgery, the Central Hospital of Lishui City, Lishui, China; ^5^ Department of General Surgery, Shengzhou People’s Hospital, Shengzhou, China; ^6^ Department of Abdominal Surgery, GuangFu Hospital, Jinhua, China

**Keywords:** gastric cancer, conversion surgery, apatinib, safety, unresectable abdominal neoplasms

## Abstract

**Objective:** Conversion therapy (surgical resection after chemotherapy) is a promising option for unresectable gastric cancer (GC) patients. Addition of anti-angiogenesis drug improves response to chemotherapy. Hence, this study explored the feasibility and efficacy of preoperative paclitaxel (PTX)/S1 chemotherapy combined with apatinib for unresectable GC.

**Methods:** Thirty-one eligible patients with a single unresectable factor were enrolled in this multi-center, single-arm trial. Apatinib (500 mg qd) was administered continuously, while PTX (130 mg/m^2^) on day 1 and S1 (80 mg/m^2^) on day 1–14 were given every 3 weeks. The treatment was given for three cycles preoperatively, but the last cycle did not include apatinib. The primary objective measurements included R0 resection rate, objective response rate (ORR) and morbidity of preoperative treatment.

**Results:** Among the 31 patients, 30 patients were evaluable for tumor response, the ORR to preoperative treatment was 73.3%. Eighteen of 30 patients underwent surgery, and R0 resection was achieved in 17 patients. The patients who underwent the conversion surgery had a superior OS compared with those who did not (3 years OS: 52.9 vs 8.3%, *p* = 0.001). The surgery was operated after apatinib had stopped for a median duration of 4 weeks. Neither anastomotic leakage nor wound healing complications was observed. No increased bleeding event was observed compared with historical data. During preoperative treatment, grade 3 or 4 toxicities were experienced by 58.1% of the patients.

**Conclusion:** Chemotherapy in combination with apatinib demonstrated higher rates of conversion and R0 resection and a superior survival benefit in initial unresectable GC. It is safe and reasonable to suspend apatinib for 4 weeks before the gastrectomy.

## Introduction

Gastric cancer is a common carcinoma which has been estimated to take up one-third of cancer-related deaths (Torre et al., 2015). Surgery is the optimal treatment for patients with GC. However, the disease is often asymptomatic at early stage and always diagnosed in later stages characterized by invasion or metastasis (Hu et al., 2020). The prognosis of unresectable GC is very poor, even targeted therapy for human epidermal growth factor receptor-2 (HER-2) makes an improved overall survival (OS) of less than 16 months (Bang et al., 2010; Shen et al., 2013).

Growing evidence has suggested that combined chemotherapy occasionally allows conversion of an initially unresectable GC to a resectable cancer. Curative, rather than palliative, resection of both primary and metastatic lesions based on the response to chemotherapy may result in long-term survival in selected patients. This treatment strategy is currently defined as conversion surgery (Yoshida et al., 2016). A large retrospective study on 151 cases found that chemotherapy followed by conversion surgery prolonged the OS compared with chemotherapy alone (53 *vs*. 14 months) (Fukuchi et al., 2015).

For the preoperative treatment, the addition of targeted drugs to chemotherapy regimens has been recognized as an effective method to improve both response rates and survival outcomes in advanced GC patients. The ToGA trial demonstrated that addition of trastuzumab to chemotherapy showed an advantage in terms of response rate and OS for patients with HER-2-positive GC tumors (Bang et al., 2010). Unfortunately, only a small percentage of patients (approximately 20%) are ideal candidates for HER-2-targeted treatment (Bang et al., 2010; Janjigian et al., 2012). Targeting vascular endothelial growth factor (VEGF) is considered as a potential treatment due to the characteristic features of VEGF expression in GC (Cancer Genome Atlas Research Network, 2014). A phase III trial recently confirmed that apatinib, the first oral tyrosine kinase inhibitor selectively targeting VEGFR-2, significantly improved the OS and progression-free survival (PFS) with an acceptable safety profile in advanced or metastatic GC patients, who had undergone at least two lines of prior chemotherapy (Li et al., 2016). Furthermore, apatinib exhibited synergistic anti-tumor effects with paclitaxel (PTX) and 5-Fu in GC cells and xenograft models (Cheng et al., 2017; Feng and Qin, 2018). In this context, apatinib plus chemotherapy might be an appealing option in conversion surgery for patients with initially unresectable GC. Thus, we conducted this multi-institutional trial to evaluate the safety and efficacy of PTX plus S1 chemotherapy in combination with apatinib (defined as SPA treatment) as preoperative treatment for GC with a single unresectable factor.

## Methods

### Patients

This study was conducted as a prospective multi-institutional trial between 2015 and 2016 at five institutions. This trial was approved by the Ethics Review Committee of Zhejiang Cancer Hospital, the First Affiliated Hospital of Zhejiang Chinese Medical University, the Central Hospital of Lishui City, Shengzhou People’ Hospital and Guangfu Hospital. All enrolled patients provided written informed consent before the study entry. This trial was registered on the ClinicalTrials network (http://www.clinicaltrial.gov) as NCT02529878 (August 20, 2015).

Patients with advanced HER-2 negative gastric adenocarcinoma with a single initially unresectable factor were recruited in this trial. The detailed eligibility criteria are summarized in Supplementary Table S1. A single unresectable factor was defined as follows: 1) multiple hepatic metastases (H1) < 3, and each tumor size <5 cm; 2) extensive lymph node metastases (ELM) defined as para-aortic lymph node (PAN) metastasis or bulky lymph nodes along the celiac, splenic, common, or proper hepatic arteries, or both of these features (Ito et al., 2017); or 3) peritoneal metastasis (CY1, P1). Staging laparoscopy was conducted before the entry into the study.

### Preoperative SPA Treatment and Assessment

As mentioned in the previous article (Xu et al., 2019a), apatinib was administered at a dose of 500 mg once a day continuously. PTX at a dose of 130 mg/m^2^ was given on day 1 as a 2 h intravenous infusion with standard premedication. Besides intravenous PTX at 60 mg/m^2^ on day 1 on schedule, patients with peritoneal metastasis received an extra intraperitoneal PTX at 70 mg/m^2^. S1 was administered consecutively at daily dose of 80 mg/m^2^ for 14 days, followed by 7 days’ off. The treatment was administered for three 21 days cycles before surgery, whereas apatinib was not included in the third cycle for the sake of safety (Supplementary Figure S1).

Toxicity was determined per the Common Terminology Criteria for Adverse Events, version 4.0 (US Department of Health Human Services, 2009). The clinical response was assessed after three cycles of preoperative treatment based on contrast-enhanced computed tomography (CT) or magnetic resonance imaging (MRI) findings in accordance with the New Response Evaluation Criteria in Solid Tumors: revised RECIST guidelines (version 1.1) (Eisenhauer et al., 2009). The assessed clinical effects included complete response (CR), partial response (PR), stable disease (SD), and progressive disease (PD).

### Conversion Surgery and Assessment

The indication for surgery was the possibility of curative resection on the basis of the response to combination treatment of apatinib and chemotherapy. A multidisciplinary team consisting of experienced medical oncologists, surgeons, and radiologists confirmed whether the patient was eligible for conversion gastrectomy. Laparotomic curative gastrectomy with D2 or more lymph node dissection, plus metastasectomy if needed, were performed.

Surgical complications were assessed as per the Clavien–Dindo classification (Katayama et al., 2016). All resected specimens were examined by the same pathologists to evaluate the extent of residual disease, disease stage, and efficacy of preoperative treatment on the basis of the Japanese Classification of Gastric Carcinoma (JCGC) third edition criteria (Japanese Gastric Cancer Association, 2011). Tumor response was graded according to the degree of tumor necrosis or disappearance relative to the estimated total number of lesions. Tumor regression was divided into five classes as follows: grade 0, no evidence of effect; grade 1a, <1/3 lesions affected; grade 1b, ≥1/3 and <2/3 affected; grade 2, ≥2/3 affected; and grade 3, no viable tumor cells remained.

Postoperative adjuvant treatment with apatinib and S1 was restarted 4 weeks after surgery for another three cycles.

### Statistical Analysis

Continuous variables are expressed as median or mean and range. Kaplan-Meier method is used to calculate the cumulative OS rates. All statistical analyses were performed with SPSS 23.0, and *p* values of 0.05 were considered as statistically significant.

## Results

### Patients Characteristics

**FIGURE 1:**
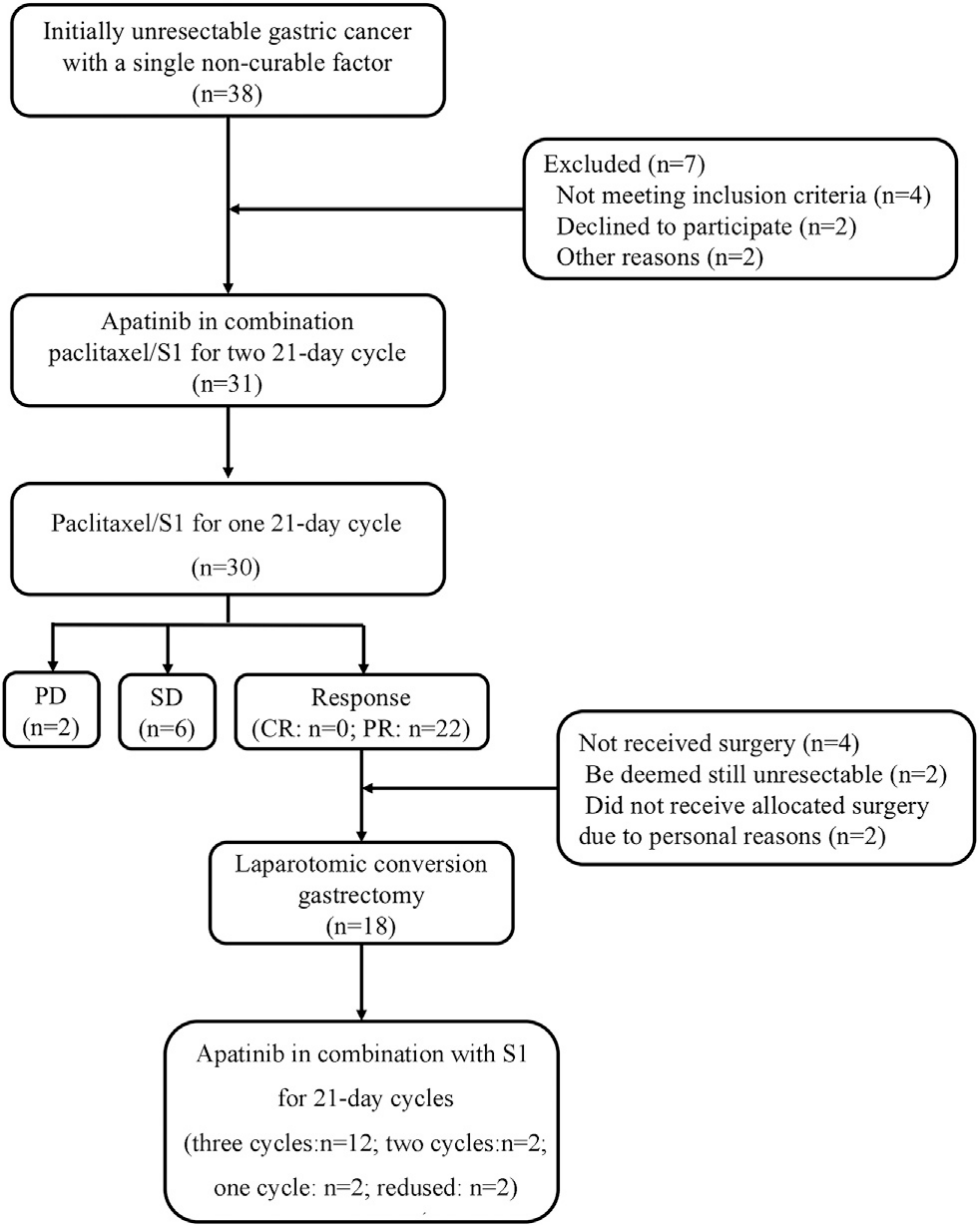
Flow diagram.

From October 2015 to December 2016, 31 patients with unresectable factors were enrolled into this study. The study flow diagram is shown in Figure 1. Patient characteristics are summarized in Table 1. Thirty-eight patients who had initially unresectable gastric cancer with a single non-curable factor was screened, and among them, seven patients were excluded because of different reasons. Thirty-one patients completed apatinib in combination paclitaxel/S1 for three 21 days cycles. And 30 patients were evaluated for tumor response. No CR was observed. PR was achieved in 22 patients, while SD was achieved in six patients. The remaining two patients developed PD. Among the 22 patients with PR response, 18 patients received surgery. The median age was 60 (34–69) years old. 51.6% (16/31) of patients had Zubrod- Eastern Cooperative Oncology Group-World Health Organization (Zubrod-ECOG-WHO) performance status of 1, and 48.4% (15/31) had ECOG performance status 0. A large proportion of patients had poorly differentiated adenocarcinoma (35.5%). As for the incurable factors, 18 (58.1%) had ELM, 9 (29.0%) had peritoneal metastasis, and 4 (12.9%) had liver metastasis.

**TABLE 1:** Patient characteristics.

Parameter	No. (31)	%
Median age (range)	60 (34–69)	
Gender		
Male	19	61.3%
Female	12	38.7%
ECOG performance status		
0	15	48.4%
1	16	51.6%
Location of primary tumor		
Upper third	7	22.6%
Middle third	7	22.6%
Lower third	13	41.9%
Diffused	4	12.9%
Non-curable factors		
Extensive lymph node metastasis	18	58.1%
Liver metastasis	4	12.9%
Peritoneal metastasis	9	29.0%
Histologic differentiation		
Moderately differentiated adenocarcinoma	1	3.2%
Poorly differentiated adenocarcinoma	11	35.5%
Poorly differentiated and signet-ring-cell carcinoma	6	19.4%
Signet-ring-cell carcinoma	2	6.5%
Unknown	11	35.5%
cTNM (31)		
III	8	25.8%
IVA	9	29.0%
IVB	13	41.9%
Unknown	1	3.2%
ypTNM(18)		
II	1	5.6%
III	10	55.6%
IV	6	33.3%
Unknown	1	5.6%

### Preoperative Treatment and Toxicity

Thirty patients completed three cycles of preoperative treatment while one patient withdrew before the scheduled preoperative evaluation due to toxicity. Twenty-three patients received full-dose chemotherapy, while seven patients required dose reductions, including 4 with a dose reduction in PTX and three in both S1 and PTX. Treatment administration was delayed in four patients due to neutropenia (*n* = 1), hematemesis (*n* = 1), colonic obstruction (*n* = 1) and oral mucositis (*n* = 1). Apatinib administration was interrupted in three patients for 4, 5 and 6 days, and no dose reduction was required.

The incidence of adverse events (AEs) of any grade was 93.5%, and 58.1% patients experienced AEs of grade 3 or 4. The most common grade 3 or higher hematologic toxicities included neutropenia (*n* = 6, 19.4%) and leukopenia (*n* = 5, 16.1%), whereas the most common non-hematological AEs were hyperbilirubinemia (*n* = 5, 16.1%), oral mucositis (*n* = 2, 6.5%), fatigue (*n* = 2, 6.5%) and hypertension (*n* = 1, 3.2%) (Table 2). A low incidence of rare and serious AEs that may be associated with apatinib included leukopenia (*n* = 1, 3.2%) and upper gastrointestinal hemorrhage (*n* = 1, 3.2%). No grade 3/4 proteinuria was observed.

**TABLE 2:** Incidence of adverse events during preoperative treatment.

Adverse events	Any grade, n (%)	Grade 3/4, n (%)
Hematological		
Leukopenia	14 (45.2%)	5 (16.1%)
Neutropenia	14 (45.2%)	6 (19.4%)
Hemoglobinopenia	13 (41.9%)	2 (6.5%)
Granulocytopenia	5 (16.1%)	3 (9.7%)
Thrombocytopenia	5 (16.1%)	1 (3.2%)
Myelopenia	2 (6.5%)	2 (6.5%)
Anemia	2 (6.5%)	0 (0%)
Non-hematological		
Hyperbilirubinemia	12 (38.7%)	5 (16.1%)
Oral mucositis	12 (38.7%)	2 (6.5%)
Fatigue	9 (29.0%)	2 (6.5%)
Nausea	6 (19.4%)	1 (3.2%)
Hand-foot syndrome	6 (19.4%)	1 (3.2%)
Hypertension	6 (19.4%)	1 (3.2%)
Proteinuria	5 (16.1%)	0 (0%)
Hypocalcaemia	4 (12.9%)	0 (0%)
Vomiting	4 (12.9%)	0 (0%)
transaminase elevated	4 (12.9%)	0 (0%)
Gastrointestinal reaction	4 (12.9%)	0 (0%)
Stomachache	2 (6.5%)	1 (3.2%)
Hypophosphatemia	2 (6.5%)	1 (3.2%)
Gastrointestinal hemorrhage	2 (6.5%)	1 (3.2%)
Diarrhea	2 (6.5%)	0 (0%)
Hypoproteinemia	2 (6.5%)	0 (0%)
Fecal occult blood	2 (6.5%)	0 (0%)
Sore throat	2 (6.5%)	0 (0%)
Headache	2 (6.5%)	0 (0%)
Cardiac failure	1 (3.2%)	1 (3.2%)
Colonic obstruction	1 (3.2%)	1 (3.2%)
Hematemesis	1 (3.2%)	1 (3.2%)
Hyperglycemia	1 (3.2%)	0 (0%)
Hyperkalemia	1 (3.2%)	0 (0%)
Liver dysfunction	1 (3.2%)	0 (0%)
Alanine aminotransferase increased	1 (3.2%)	0 (0%)
Alkaline phosphatase increased	1 (3.2%)	0 (0%)
Epistaxis	1 (3.2%)	0 (0%)
Hematuria	1 (3.2%)	0 (0%)
Activated partial thromboplastin time prolonged	1 (3.2%)	0 (0%)
Pain	1 (3.2%)	0 (0%)
Decreased albumin	1 (3.2%)	0 (0%)

### Efficacy of the Preoperative Treatment

Total 30 patients were evaluated for tumor response in Table 3. No CR was observed. PR was achieved in 22 patients, while SD was achieved in six patients. Thus, the ORR was 73.3% (22/30), and the disease control rate (DCR) was 93.3% (28/30).

**TABLE 3:** Primary assessment method: Overall assessment.

Parameter	N
Number of patients screened	38
Number of patients enrolled	31
Number of patients completed three 21-days cycle combination treatment	30
Number of patients evaluated toxicity	31
Number of patients evaluated tumor response	30
Number of patients received surgery	18
Evaluation method	RECIST 1.1
Response assessment CR	N = 0 (0)
Response assessment PR	N = 22 (73.3%)
Response assessment SD	N = 6 (20%)
Response assessment PD	N = 2 (6.7%)
Response assessment ORR	N = 28 (73.3%)
Response assessment DCR	N = 2 (6.7%)
R0 resection	N = 17 (56.7%)

### Surgical Outcome

Among the 22 patients who achieved PR after SPA treatment, two patients were deemed still unresectable by the multidisciplinary team due to ascites and extensive involvement of surrounding structures, two patients refused the predesigned surgery for personal reasons, and thus 18 patients underwent surgery eventually. The median duration from the last administration of apatinib and S1 to surgery was 29.4 ± 2.9 days and 15.5 ± 2.9 days, respectively. Among the 18 patients, 17 underwent R0 resection, and one underwent R1 resection (for positive surgical margins). Pathological responses (≥grade 1b) were observed in 11 patients in primary lesions (Table 4). Complete pathologic responses, (i.e., no identifiable viable tumor cells) were observed in three patients. In metastatic tumors, the degree of pathologic response was more significant. Five patients who had metastases at diagnosis and received metastasectomy after SPA treatment were considered to have complete pathological responses in their metastases. Table 4 summarizes the surgical and pathological information for these patients.

**TABLE 4:** Surgical and pathological details.

Parameter	No. (18)	%
Depth of tumor invasion		
ypT1	1	5.6
ypT2	1	5.6
ypT3	0	0
ypT4	16	88.8
Nodal status		
ypN0	7	38.9
ypN1	5	27.8
ypN2	2	11.1
ypN3	4	22.2
Non-curable factors		
Extensive lymph node metastasis	13	72.2
Liver metastasis	2	11.1
Peritoneal metastasis	3	16.7
Type of gastric resection		
Total gastrectomy	14	77.8
Distal gastrectomy	4	22.2
Lymph node dissection		
D2	15	83.3
D2+	3	16.7
Resected adjacent organs		
No	12	66.7
Yes	6	33.3
Blood loss (ml), median (range)	150 (50–500)	
Pathological response (primary tumor)		
Grade 0	0	0
Grade 1a	3	16.7
Grade 1b	3	16.7
Grade 2	5	27.8
Grade 3	3	16.7
Not evaluated	4	22.2
Postoperative complication	None	

The median duration of postoperative hospitalization was 12.1 ± 3.2 days. Fifteen complications were observed in 18 patients who underwent gastrectomy, 11 grade I (not requiring special treatment), 3 grade II (requiring special treatment such as a blood transfusion), 1 grade IIIa (requiring surgical, endoscopic or radiological intervention without anesthesia) (Xu et al., 2019b), and no grade IIIb or higher complications. All complications were resolved with conservative treatment. No case of re-operation was reported. None of the anastomotic leakage, wound healing complications, or increased bleeding events was observed.

Twelve of 18 patients completed three cycles of adjuvant treatment using the recommended regimen, S1 plus apatinib, according to this protocol. Two patients received two cycles and one patient received one cycle of adjuvant treatment; three patients refused adjuvant therapy.

### Three-Year Overall Survival

**FIGURE 2:**
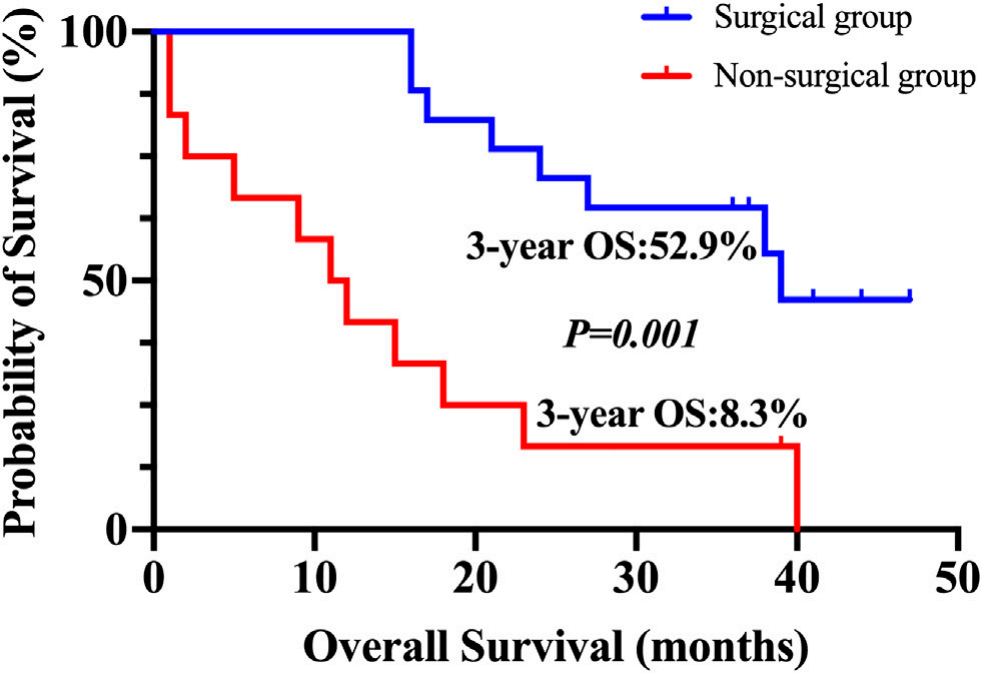
Kaplan–Meier plot for 3-years overall survival. The overall survival for patients who underwent conversion surgery and for those who did not.

At the cut-off date of June 2019, two of 30 patients lost follow-up, including one patient who underwent the conversion surgery and one patient who did not. The patients who underwent the conversion surgery had a superior OS compared with those who did not (*p* = 0.001). For subjects who received the conversion therapy, the rate of 3 years OS was 52.9%. Nevertheless, patients who were not eligible for the conversional therapy, the 3 years OS survival rate was 8.3% (Figure 2).

## Discussion

To the best of our knowledge, this was the first trial to explore the safety and efficacy of apatinib combined with chemotherapy for conversional therapy in GC patients. The results showed that after preoperative treatment with apatinib plus PTX and S-1 chemotherapy (PS regimen), the ORR was 73.3% and DCR was 93.3%, the conversion rate and R0 resection rate achieved 60.0 and 56.7%, respectively. Pathological responses (≥grade 1b) were observed in 11 patients in primary lesions, including complete pathological response observed in three patients. For patients received conversional therapy, the rate of 1-year OS was 100% and the rate of 3 years OS was 52.9%.

As for conversion therapy, it hasn’t been recognized internationally. When it comes to “unresectable,” the situation is more varied. Terashima suggested that cases of T4b tumor and distant lymph node metastasis were good candidates for conversion therapy (Terashima, 2016). Yoshida et al., classified stage IV cases into four categories, and suggested the best candidates for conversion therapy were patients with marginally resectable metastasis without peritoneal dissemination and part of patients with peritoneal metastasis (Yoshida et al., 2016). In this study, the eligible patients with unresectable factors were determined by the multidisciplinary team according to the criteria from Fujitani, including 18 patients with extensive lymph node metastasis, nine patients with peritoneal metastasis, and four patients with hepatic metastasis.

Several studies about conversion therapy were primarily carried out in Japan. The commonly used chemotherapy regimen for GC was S-1 plus cisplatin regimen, capecitabine plus cisplatin (XP) regimen and S-1 plus docetaxel regimen. PTX showed innate anti-angiogenic properties through directly mediating endothelial cell function and inhibiting of VEGF-induced angiogenesis *in vivo* and *vitro* (Hu et al., 2019). It might be attributed to the synergy of PTX and ramucirumab resulting in an improved survival shown in the RAINBOW trial. Since apatinib also targets VEGFR-2 and regulates its mediated signaling pathway, the combination of apatinib and PTX-based chemotherapy may have a synergistic effect. Furthermore, PS regimen has been regarded as a hopeful regimen (Cancer Genome Atlas Research Network, 2014). So in this study, the regimen of apatinib combined with PTX plus S-1 was used. The feasibility and effectiveness of preoperative apatinib in combination with PS chemotherapy were explored. The results showed that for the 30 patients evaluated, 22 patients achieved PR, and six patients achieved SD, the ORR (the primary endpoint of this study) was 73.3%, and the DCR was 93.3%.

In this study, the conversion rate was 60%, and the R0 resection rate was 56.7%. In two retrospective studies in Japan which enrolled similar unresectable patients as in this study, the R0 resection rate were similar. The conversion rate and R0 resection rate are affected by the type of unresectable factors. Compared with tumor invasion of adjacent structures and severe lymph node metastases (Inoue et al., 2012; Yamaguchi et al., 2018), peritoneal metastasis or positive peritoneal cytology findings (P1/CY1) caused a lower conversion rate and R0 resection rate. So, the enrolled patients with different type of unresectable factors might could explain the variance between our study and other studies in R0 resection rate and clinical outcome.

Our results revealed that the combination of apatinib and PS chemotherapy did not substantially alter the safety profile of chemotherapy. The most common grade 3 or 4 AEs was neutropenia, which occurred in 19.4% of the patients. The incidence of hematologic AEs was consistent with previous studies of PS. However, one patient died from chemotherapy-induced myelosuppression. In the treatment-related death from SPA treatment, severe myelosuppression appeared after the second cycle of administration, leading to fatal infection and heart failure. In addition, the rate of AEs associated with apatinib, including hypertension and proteinuria, was low, and these events were effectively managed. No other classical toxicities associated with anti-angiogenic drugs, particularly bleeding, gastrointestinal perforation, or venous thromboembolism, was observed during preoperative treatment. In summary, the safety profile of apatinib in combination with PS regimen in this study was acceptable, whereas the severe hematological toxicity should be managed carefully, especially during the first cycle of treatment.

Previous studies about bevacizumab demonstrated that anti-angiogenic agents should be better discontinued at least 5–8 weeks prior to surgery and not be restarted until 28 days after the surgery in the setting of neoadjuvant and conversion therapy of metastatic colorectal cancer (Gruenberger et al., 2008; Xu et al., 2019a). Our data confirmed that, the duration of withdrawal apatinib for 4 weeks, the interval between radical gastrectomy and the last dose of apatinib, did not increase the rate of complications in metastatic GC patients. Compared with our historical data, intraoperative hemorrhage didn’t increase, neither did delayed wound healing complications nor anastomotic dehiscence occurred. These results showed that the addition of apatinib to the chemotherapy did not affect perioperative morbidity under appropriate management.

Although this study is limited by a prespecified sample size and relatively short follow-up period, apatinib combined with PS chemotherapy seem to be highly active against unresectable GC, allowing conversional resection with curative intent in 60% of patients.

## Conclusion

Conversion therapy is a new concept and recognized as a potential strategy for improving expectancy in patients with advanced and recurrent cancer. Apatinib plus PS chemotherapy followed by conversional resection may be a feasible, safe, and new option for initially unresectable GC. Large scaled randomized controlled trials are needed to further evaluate the efficacy of apatinib plus PS chemotherapy in conversion therapy in GC patients.

## Data Availability

The original contributions presented in the study are included in the article/Supplementary Material, further inquiries can be directed to the corresponding author.
