# Supplementary material for: Efficacy of Conversion Surgery Following Apatinib Plus Paclitaxel/S1 for Advanced Gastric Cancer With Unresectable Factors: A Multicenter, Single-Arm, Phase II Trial
Source: Front Pharmacol. 2021 Mar 19;12:642511. doi: 10.3389/fphar.2021.642511 (PMC8017219; doi:10.3389/fphar.2021.642511)
Supplement: Supplementary file 1 [file datasheet1.docx]

**Supplementary Figure S1: Treatment strategy**


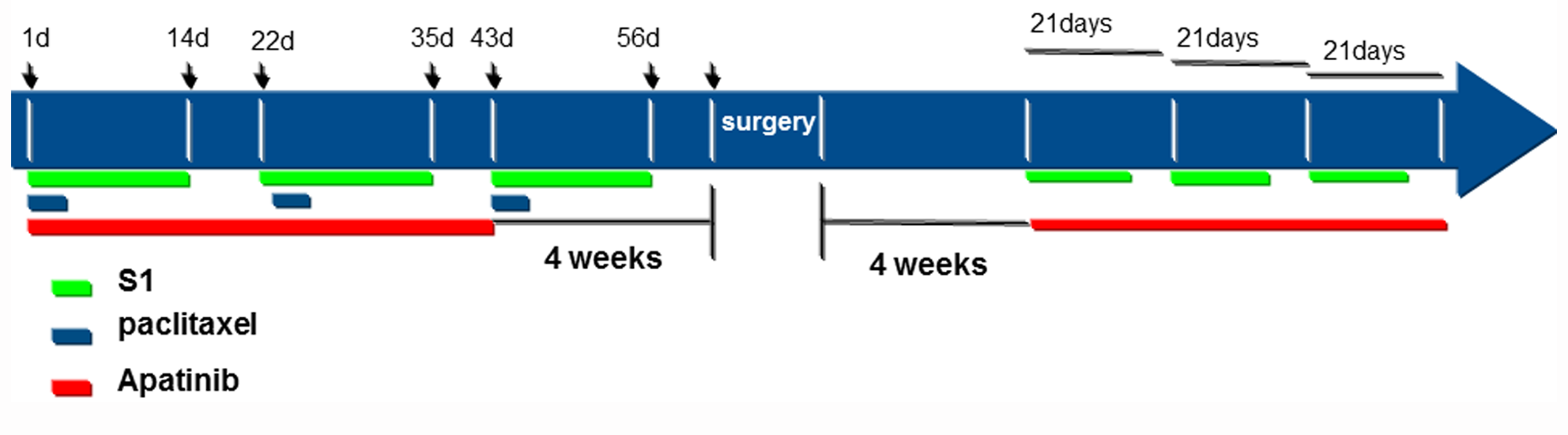


The subsequent chemotherapy cycle was delayed for up to 1 week if the neutrophil count was lower than 1,500/mm^3^, the platelet count was lower than 75,000/mm^3^, aspartate aminotransferase and alanine aminotransferase levels were higher than 100 IU/L, or the serum creatinine concentration was higher than 1.5 mg/dL. The dose of PTX and/or S1 was reduced by 25% for all subsequent cycles when patients developed a grade 4 hematologic and/or grade 3 or higher non-hematologic adverse events. Treatment interruption and/or dose reduction to 250 mg qd were allowed in case of grade 3 hematologic and/or grade 2 or higher nonhematologic toxicities.

**Supplementary Table S1: Eligibility criteria**

| - Inclusion criteria | - Exclusion criteria |
| --- | --- |
| Histopathologically confirmed gastric adenocarcinoma. | With distant metastasis in lung, brain, bone, etc., except for liver. |
| Had a single unresectable factor suggested by preoperative examinations including CT, MRI or PET-CT. | Her-2 positive and willing to receive trastuzumab treatment. |
| Preoperative laparoscopic surgery had defined the diagnosis. | With other unmanageable serious diseases (including atrial fibrillation, angina, cardiac insufficiency, ejection fraction < 50%, refractory hypertension, renal dysfunction). |
| No prior treatment (such as radiotherapy, chemotherapy, targeted therapy, and immunotherapy). | Had received other medical treatment (including traditional Chinese medicine) or can not guarantee to follow the research as requested after enrollment. |
| ECOG (Eastern Cooperative Oncology Group) Score: 0-1. | Allergic to drugs in this protocol. |
| Age between 18 and 70 years; life expectancy >3 months. | Pregnant and lactating women. |
| Hemodynamic parameters were normal before enrollment (including blood cell counts, hepatic and renal function), such as WBC>4.0×10^9^/L, neutrophil >1.5×10^9^/L, platelet >100×10^9^/L bilirubin<1.5 times as the upper limit of normal range, AST, ALT, Cr <2.5 times as the upper limit of normal range, SCr <1.2 mg/dl. | Women of child-bearing age who had procreation demand during the research. |
| Cardiac function was normal before enrollment. No myocardial infarction attack within half a year. Hypertension, coronary heart diseases were under control at the time of enrollment. |  |
| Without other uncontrollable benign diseases, such as pulmonary diseases, renal diseases, hepatopathy, and infections. |  |
| Not participate in other research projects before and during treatment. |  |
